# Supplementary material for: RNA structure drives interaction with proteins
Source: Nat Commun. 2019 Jul 19;10:3246. doi: 10.1038/s41467-019-10923-5 (PMC6642211; doi:10.1038/s41467-019-10923-5)
Supplement: Supplementary file 1 — Supplementary Information [file 41467_2019_10923_MOESM1_ESM.pdf]

Supplementary Information

RNA structure drives interaction with proteins

Natalia Sanchez de Groot[1,*], Alexandros Armaos[1,*], Ricardo Graña-Montes[1,2], Marion Alriquet[3,4] , Giulia Calloni[3,4], R. Martin Vabulas[3,4,**], and Gian Gaetano Tartaglia[1,5,6,7 **]

[1] Centre for Genomic Regulation (CRG), The Barcelona Institute for Science and Technology, Dr. Aiguader 88, 08003 Barcelona, Spain

[2] Current address: Department of Biochemistry, University of Zürich. Winterthurerstrasse 190, 8057, Zürich, Switzerland.

[3] Buchmann Institute for Molecular Life Sciences, Goethe University Frankfurt, 60438 Frankfurt am Main, Germany.

[4] Institute of Biophysical Chemistry, Goethe University Frankfurt, 60438 Frankfurt am Main, Germany

[5] ICREA 23 Passeig Lluis Companys 08010 and Universitat Pompeu Fabra (UPF) 08003, Barcelona, Spain

[6] Department of Biology 'Charles Darwin', Sapienza University of Rome, P.le A. Moro 5, Rome 00185, Italy

[7] Department of Neuroscience and Brain Technologies, Istituto Italiano di Tecnologia, Via Morego 30, 16163, Genoa, Italy.

** Equal contribution

** Correspondence to RMV vabulas@em.uni-frankfurt.de and GGT gian.tartaglia@crg.eu

INDEX

Sequential alignment between HBG1 and HBG2 …………..…………..…………..……….…………2
Sequences of Braf (low structured RNA) and HSP70 (high structured RNA) …………………………3

Supplementary Figures

Supplementary Figure 1. catRAPID comparison with RPIseq. …………………...…..……………….. 4
Supplementary Figure 2. HBG1 and HBG2 sequential and structural differences ……………………. 5
Supplementary Figure 3. RNA structural content with and without UTRs 6
Supplementary Figure 4. Gene ontology for highly expressed RNAs ……………………………..…. 7
Supplementary Figure 5. Properties significantly different between ssRNA and dsRNA binders …..... 8
Supplementary Figure 6. eCLIP distribution …………………………………………………….…..... 9
Supplementary Figure 7. BioGRID vs. DMS ……………………………………………...……..10
Supplementary Figure 8. CROSS and DMS structural content for LS RNA and HS RNA ………….. 11
Supplementary Figure 9. catRAPID and eCLIP data including UTRS ……...……………………….. 12
Supplementary Figure 10. Uncropped gels from Figure 4a. …………………………...…………... 13
Supplementary Figure 11. Sets to define with catRAPID the better binders for *HSP70* ……………... 14

Supplementary Tables

Supplementary Table 1. Structural content by RNA type …………………………...……………. 15
Supplementary Table 2. Correlation of different scales of hydrophobicity with polarity ……………. 16
Supplementary Table 3. Common RNA-binding domains ……………………...………………..… 17

Supplementary References …..……………………………………………………………..…… 18

Sequential alignment between HBG1 and HBG2

```
HBG1   1     ACACTCGCTTCTGGAACGTCTGAGGTTATCAATAAGCTCCTAGTCCAGACGCCATGGGTC  60
             ||||||||||||||||||||||||||||||||||||||||||||||||||||||||||||
HGB2   1     ACACTCGCTTCTGGAACGTCTGAGGTTATCAATAAGCTCCTAGTCCAGACGCCATGGGTC  60

HBG1   61    ATTTCACAGAGGAGGACAAGGCTACTATCACAAGCCTGTGGGGCAAGGTGAATGTGGAAG  120
             ||||||||||||||||||||||||||||||||||||||||||||||||||||||||||||
HGB2   61    ATTTCACAGAGGAGGACAAGGCTACTATCACAAGCCTGTGGGGCAAGGTGAATGTGGAAG  120

HBG1   121   ATGCTGGAGGAGAAACCCTGGGAAGGCTCCTGGTTGTCTACCCATGGACCCAGAGGTTCT  180
             ||||||||||||||||||||||||||||||||||||||||||||||||||||||||||||
HGB2   121   ATGCTGGAGGAGAAACCCTGGGAAGGCTCCTGGTTGTCTACCCATGGACCCAGAGGTTCT  180

HBG1   181   TTGACAGCTTTGGCAACCTGTCCTCTGCCTCTGCCATCATGGGCAACCCCAAAGTCAAGG  240
             ||||||||||||||||||||||||||||||||||||||||||||||||||||||||||||
HGB2   181   TTGACAGCTTTGGCAACCTGTCCTCTGCCTCTGCCATCATGGGCAACCCCAAAGTCAAGG  240

HBG1   241   CACATGGCAAGAAGGTGCTGACTTCCTTGGGAGATGCCACAAAGCACCTGGATGATCTCA  300
             ||||||||||||||||||||||||||||||||||||||| ||||||||||||||||||||
HGB2   241   CACATGGCAAGAAGGTGCTGACTTCCTTGGGAGATGCCATAAAGCACCTGGATGATCTCA  300

HBG1   301   AGGGCACCTTTGCCCAGCTGAGTGAACTGCACTGTGACAAGCTGCATGTGGATCCTGAGA  360
             ||||||||||||||||||||||||||||||||||||||||||||||||||||||||||||
HGB2   301   AGGGCACCTTTGCCCAGCTGAGTGAACTGCACTGTGACAAGCTGCATGTGGATCCTGAGA  360

HBG1   361   ACTTCAAGCTCCTGGGAAATGTGCTGGTGACCGTTTTGGCAATCCATTTCGGCAAAGAAT  420
             ||||||||||||||||||||||||||||||||||||||||||||||||||||||||||||
HGB2   361   ACTTCAAGCTCCTGGGAAATGTGCTGGTGACCGTTTTGGCAATCCATTTCGGCAAAGAAT  420

HBG1   421   TCACCCCTGAGGTGCAGGCTTCCTGGCAGAAGATGGTGACTGCAGTGGCCAGTGCCCTGT  480
             ||||||||||||||||||||||||||||||||||||||||| ||||||||||||||||||
HGB2   421   TCACCCCTGAGGTGCAGGCTTCCTGGCAGAAGATGGTGACTGGAGTGGCCAGTGCCCTGT  480

HBG1   481   CCTCCAGATACCACTGAGCTCACTGCCCATGATTCAGAGCTTTCAAGGATAGGCTTTATT  540
             ||||||||||||||||||||||||||||||||| ||||||||||||||||||||||||||
HGB2   481   CCTCCAGATACCACTGAGCTCACTGCCCATGATGCAGAGCTTTCAAGGATAGGCTTTATT  540

HBG1   541   CTGCAAGCAATACAAATAATAAATCTATTCTGCTGAGAGATCAC  584
             ||||||||||| |||||||||||||||||||||| |||||||||
HGB2   541   CTGCAAGCAAT-CAAATAATAAATCTATTCTGCTAAGAGATCAC  583
```

Sequences of *BRaf* (low structured RNA) and *HSP70* (high structured RNA)

>BRaf

```
GCGCTGAGCGGTGGCGGTGGTGGCGGCGCGGAGCCGGGCCAGGCTCTGTTCAACGGGGACATGGAGCCCG
AGGCCGGCGCCGGCGCCGGCGCCGCGGCCTCTTCGGCTGCGGACCCTGCCATTCCGGAGGAGGTGTGGAA
TATCAAACAAATGATTAAGTTGACACAGGAACATATAGAGGCCCTATTGGACAAATTTGGTGGGGAGCAT
AATCCACCATCAATATATCTGGAGGCCTATGAAGAATACACCAGCAAGCTAGATGCACTCCAACAAAGAG
AACAACAGTTATTGGAATCTCTGGGGAACGGAACTGATTTTTCTGTTTCTAGCTCTGCATCAATGGATAC
CGTTACATCTTCTTCCTCTTCTAGCCTTTCAGTGCTACCTTCATCTCTTTCAGTTTTTCAAAATCCCACA
GATGTGGCACGGAGCAACCCCAAGTCACCACAAAAACCTATCGTTAGAGTCTTCCTGCCCAACAAACAGA
GGACAGTGGTACCTGCAAGGTGTGGAGTTACAGTCCGAGACAGTCTAAAGAAAGCACTGATGATGAGAGG
TCTAATCCCAGAGTGCTGTGCTGTTTACAGAATTCAGGATGGAGAGAAGAAACCAATTGGTTGGGACACT
GATATTTCCTGGCTTACTGGAGAAGAATTGCATGTGGAAGTGTTGGAGAATGTTCCACTTACAACACACA
ACTTTGTACGAAAAACGTTTTTCACCTTAGCATTTTGTGACTTTTGTCGAAAGCTGCTTTTCCAGGGTTT
CCGCTGTCAAACATGTGGTTATAAATTTCACCAGCGTTGTAGTACAGAAGTTCCACTGATGTGTGTTAAT
TATGACCAACTTGATTTGCTGTTTGTCTCCAAGTTCTTTGAACACCACCCAATACCACAGGAAGAGGCGT
CCTTAGCAGAGACTGCCCTAACATCTGGATCATCCCCTTCCGCACCCGCCTCGGACTCTATTGGGCCCCA
AATTCTCACCAGTCCGTCTCCTTCAAAATCCATTCCAATTCCACAGCCCTTCCGACCAGCAGATGAAGAT
CATCGAAATCAATTTGGGCAACGAGACCGATCCTCATCAGCTCCCAATGTGCATATAAACACAATAGAAC
CTGTCAATATTGATGACTTGATTAGAGACCAAGGATTTCGTGGTGATGGAGGATCAACCACAGGTTTGTC
TGCTACCCCCCCTGCCTCATTACCTGGCTCACTAACTAACGTGAAAGCCTTACAGAAATCTCCAGGACCT
CAGCGAGAAAGGAAGTCATCTTCATCCTCAGAAGACAGGAATCGAATGAAAACACTTGGTAGACGGGACT
CGAGTGATGATTGGGAGATTCCTGATGGGCAGATTACAGTGGGACAAAGAATTGGATCTGGATCATTTGG
AACAGTCTACAAGGGAAAGTGGCATGGTGATGTGGCAGTGAAAATGTTGAATGTGACAGCACCTACACCT
CAGCAGTTACAAGCCTTCAAAAATGAAGTAGGAGTACTCAGGAAAACACGACATGTGAATATCCTACTCT
TCATGGGCTATTCCACAAAGCCACAACTGGCTATTGTTACCCAGTGGTGTGAGGGCTCCAGCTTGTATCA
CCATCTCCATATCATTGAGACCAAATTTGAGATGATCAAACTTATAGATATTGCACGACAGACTGCACAG
GGCATGGATTACTTACACGCCAAGTCAATCATCCACAGAGACCTCAAGAGTAATAATATATTTCTTCATG
AAGACCTCACAGTAAAAATAGGTGATTTTGGTCTAGCTACAGTGAAATCTCGATGGAGTGGGTCCCATCA
GTTTGAACAGTTGTCTGGATCCATTTTGTGGATGGCACCAGAAGTCATCAGAATGCAAGATAAAAATCCA
TACAGCTTTCAGTCAGATGTATATGCATTTGGAATTGTTCTGTATGAATTGATGACTGGACAGTTACCTT
ATTCAAACATCAACAACAGGGACCAGATAATTTTTATGGTGGGACGAGGATACCTGTCTCCAGATCTCAG
TAAGGTACGGAGTAACTGTCCAAAAGCCATGAAGAGATTAATGGCAGAGTGCCTCAAAAAGAAAAGAGAT
GAGAGACCACTCTTTCCCCAAATTCTCGCCTCTATTGAGCTGCTGGCCCGCTCATTGCCAAAAATTCACC
GCAGTGCATCAGAACCCTCCTTGAATCGGGCTGGTTTCCAAACAGAGGATTTTAGTCTATATGCTTGTGC
TTCTCCAAAAACACCCATCCAGGCAGGGGGATATGGTGCGTTTCCTGTCCACTGA
```

>HSP70

```
ATGGCCAAAGCCGCGGCGATCGGCATCGACCTGGGCACCACCTACTCCTGCGTGGGGGTGTTCCAACACG
GCAAGGTGGAGATCATCGCCAACGACCAGGGCAACCGCACCACCCCCAGCTACGTGGCCTTCACGGACAC
CGAGCGGCTCATCGGGGATGCGGCCAAGAACCAGGTGGCGCTGAACCCGCAGAACACCGTGTTTGACGCG
AAGCGGCTGATCGGCCGCAAGTTCGGCGACCCGGTGGTGCAGTCGGACATGAAGCACTGGCCTTTCCAGG
TGATCAACGACGGAGACAAGCCCAAGGTGCAGGTGAGCTACAAGGGGGACACCAAGGCATTCTACCCCGA
GGAGATCTCGTCCATGGTGCTGACCAAGATGAAGGAGATCGCCGAGGCGTACCTGGGCTACCCGGTGACC
AACGCGGTGATCACCGTGCCGGCCTACTTCAACGACTCGCAGCGCCAGGCCACCAAGGATGCGGGTGTGA
TCGCGGGGCTCAACGTGCTGCGGATCATCAACGAGCCCACGGCCGCCGCCATCGCCTACGGCCTGGACAG
AACGGGCAAGGGGGAGCGCAACGTGCTCATCTTTGACCTGGGCGGGGGCACCTTCGACGTGTCCATCCTG
ACGATCGACGACGGCATCTTCGAGGTGAAGGCCACGGCCGGGGACACCCACCTGGGTGGGGAGGACTTTG
ACAACAGGCTGGTGAACCACTTCGTGGAGGAGTTCAAGAGAAAACACAAGAAGGACATCAGCCAGAACAA
GCGAGCCGTGAGGCGGCTGCGCACCGCCTGCGAGAGGGCCAAGAGGACCCTGTCGTCCAGCACCCAGGCC
AGCCTGGAGATCGACTCCCTGTTTGAGGGCATCGACTTCTACACGTCCATCACCAGGGCGAGGTTCGAGG
AGCTGTGCTCCGACCTGTTCCGAAGCACCCTGGAGCCCGTGGAGAAGGCTCTGCGCGACGCCAAGCTGGA
CAAGGCCCAGATTCACGACCTGGTCCTGGTCGGGGGCTCCACCCGCATCCCCAAGGTGCAGAAGCTGCTG
CAGGACTTCTTCAACGGGCGCGACCTGAACAAGAGCATCAACCCCGACGAGGCTGTGGCCTACGGGGCGG
CGGTGCAGGCGGCCATCCTGATGGGGGACAAGTCCGAGAACGTGCAGGACCTGCTGCTGCTGGACGTGGC
TCCCCTGTCGCTGGGGCTGGAGACGGCCGGAGGCGTGATGACTGCCCTGATCAAGCGCAACTCCACCATC
CCCACCAAGCAGACGCAGATCTTCACCACCTACTCCGACAACCAACCCGGGGTGCTGATCCAGGTGTACG
AGGGCGAGAGGGCCATGACGAAAGACAACAATCTGTTGGGGCGCTTCGAGCTGAGCGGCATCCCTCCGGC
CCCCAGGGGCGTGCCCCAGATCGAGGTGACCTTCGACATCGATGCCAACGGCATCCTGAACGTCACGGCC
ACGGACAAGAGCACCGGCAAGGCCAACAAGATCACCATCACCAACGACAAGGGCCGCCTGAGCAAGGAGG
AGATCGAGCGCATGGTGCAGGAGGCGGAGAAGTACAAAGCGGAGGACGAGGTGCAGCGCGAGAGGGTGTC
AGCCAAGAACGCCCTGGAGTCCTACGCCTTCAACATGAAGAGCGCCGTGGAGGATGAGGGGCTCAAGGGC
AAGATCAGCGAGGCCGACAAGAAGAAGGTGCTGGACAAGTGTCAAGAGGTCATCTCGTGGCTGGACGCCA
ACACCTTGGCCGAGAAGGACGAGTTTGAGCACAAGAGGAAGGAGCTGGAGCAGGTGTGTAACCCCATCAT
CAGCGGACTGTACCAGGGTGCCGGTGGTCCCGGGCCTGGGGGCTTCGGGGCTCAGGGTCCCAAGGGAGGG
TCTGGGTCAGGCCCCACCATTGAGGAGGTAGATTAG
```

a

10%	3289	101178
15%	4933	143112
20%	6577	184902
25%	8222	224682
30%	9866	263598
35%	11510	303435
40%	13155	341111
45%	14799	378714
50%	16444	414929

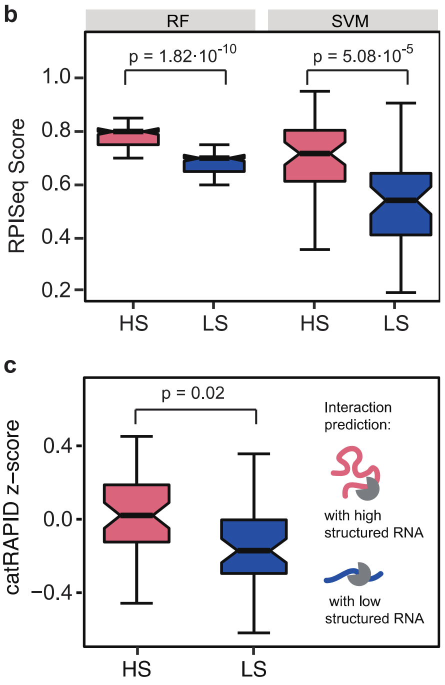

Supplementary Figure 1. RPIseq and catRAPID performances. a) Fractions (percentiles), RNAs selected for the highest-structural (HS) and lowest-structural (LS) content (equal HS and LS groups) and protein-RNA pairs reported in **Fig. 1b**. **b-c)** Interaction predictions for 50 proteins and 100 HS and LS transcripts reported in **Fig. 1b**. The boxplots show that **b)** RPIseq and **c)** catRAPID have the same trend.

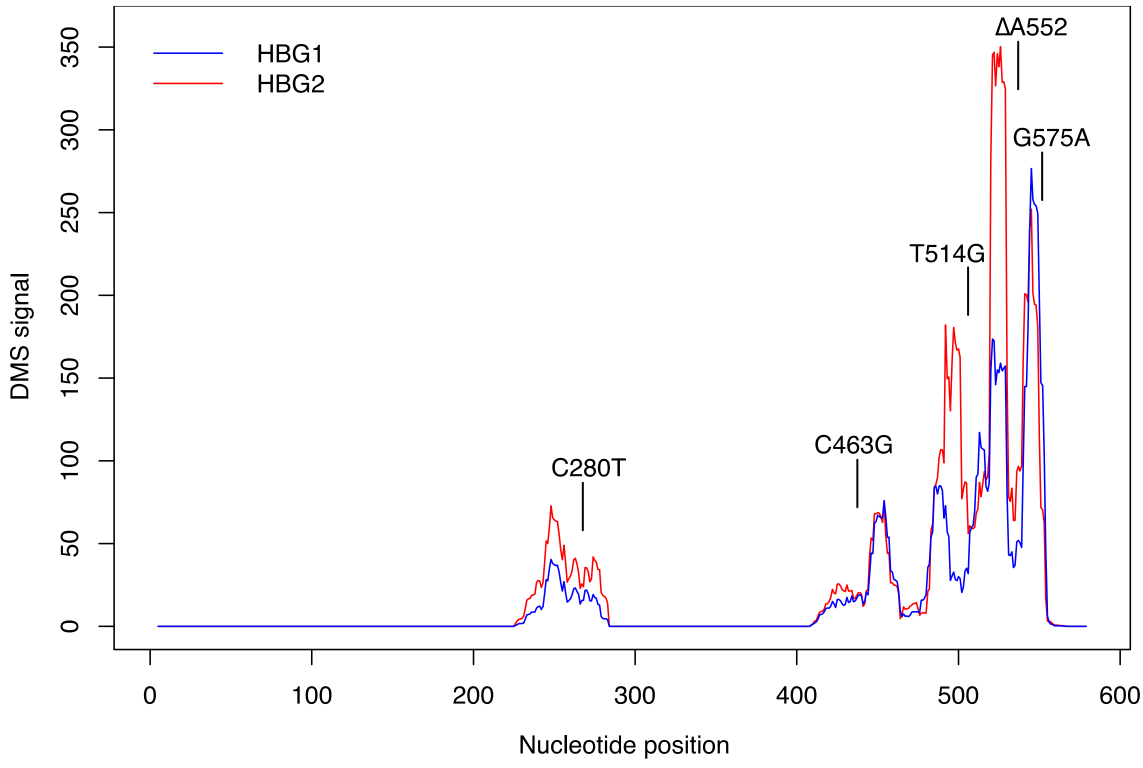

Supplementary Figure 2. HBG1 and HBG2 sequential and structural differences. Comparison between the transcripts coding for HBG1 and HBG2 gamma globins.

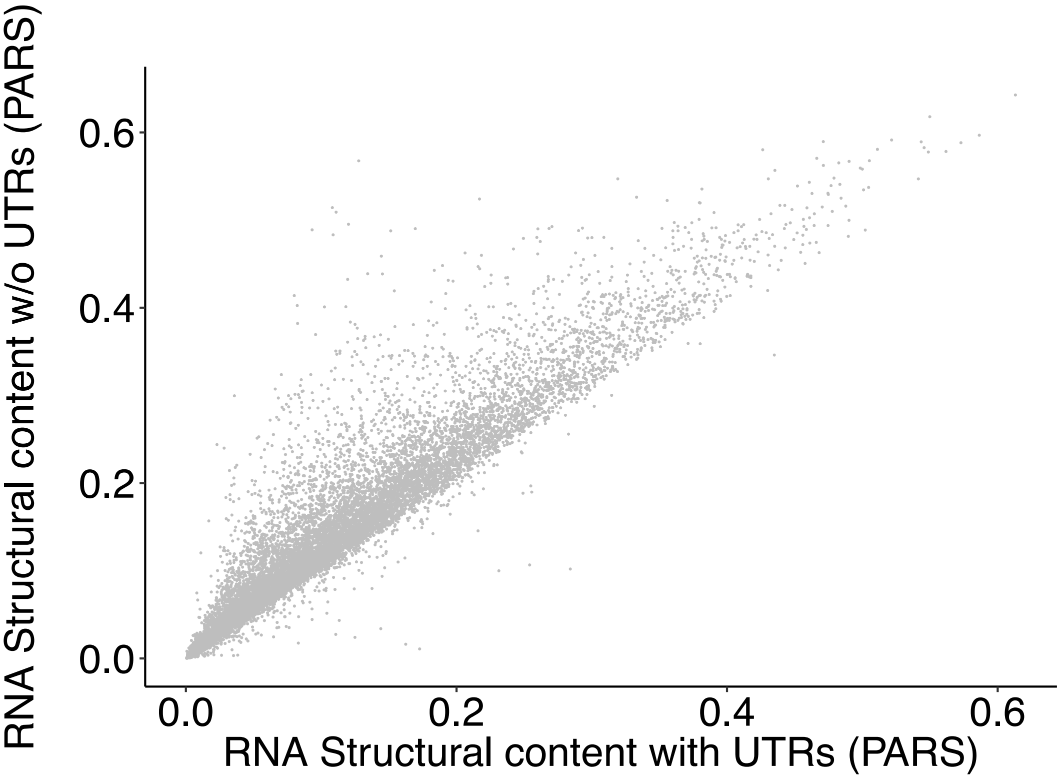

Supplementary Figure 3. Structural content per transcript with and without UTRs. We observe a transcriptome-wide correlation of 0.94 indicating that, in addition to UTRs, other regions of the RNAs are structured.

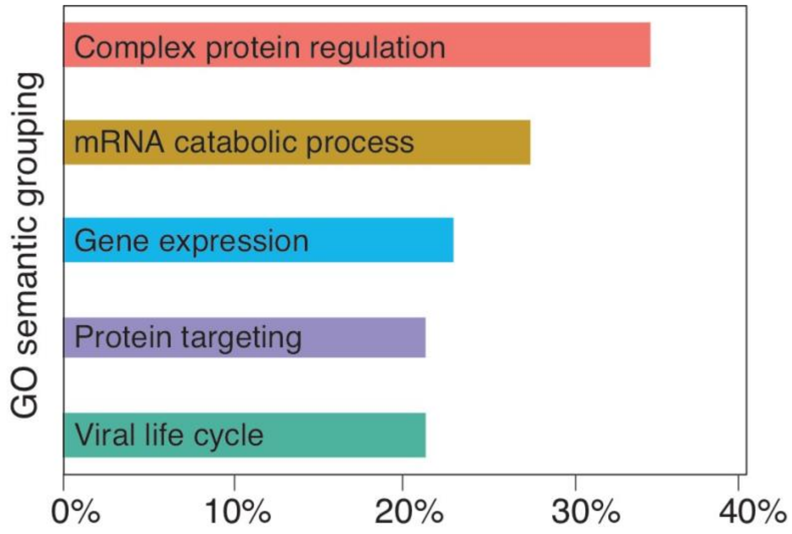

Supplementary Figure 4. Gene ontology for highly expressed RNAs. GO semantic grouping for HS against the 25% more expressed transcripts.

The data to build this image were extracted from our predictions[1] available at the webpage http://www.tartaglialab.com/GO_analyser/render_GO_universal/2500/b1c58f4a18/

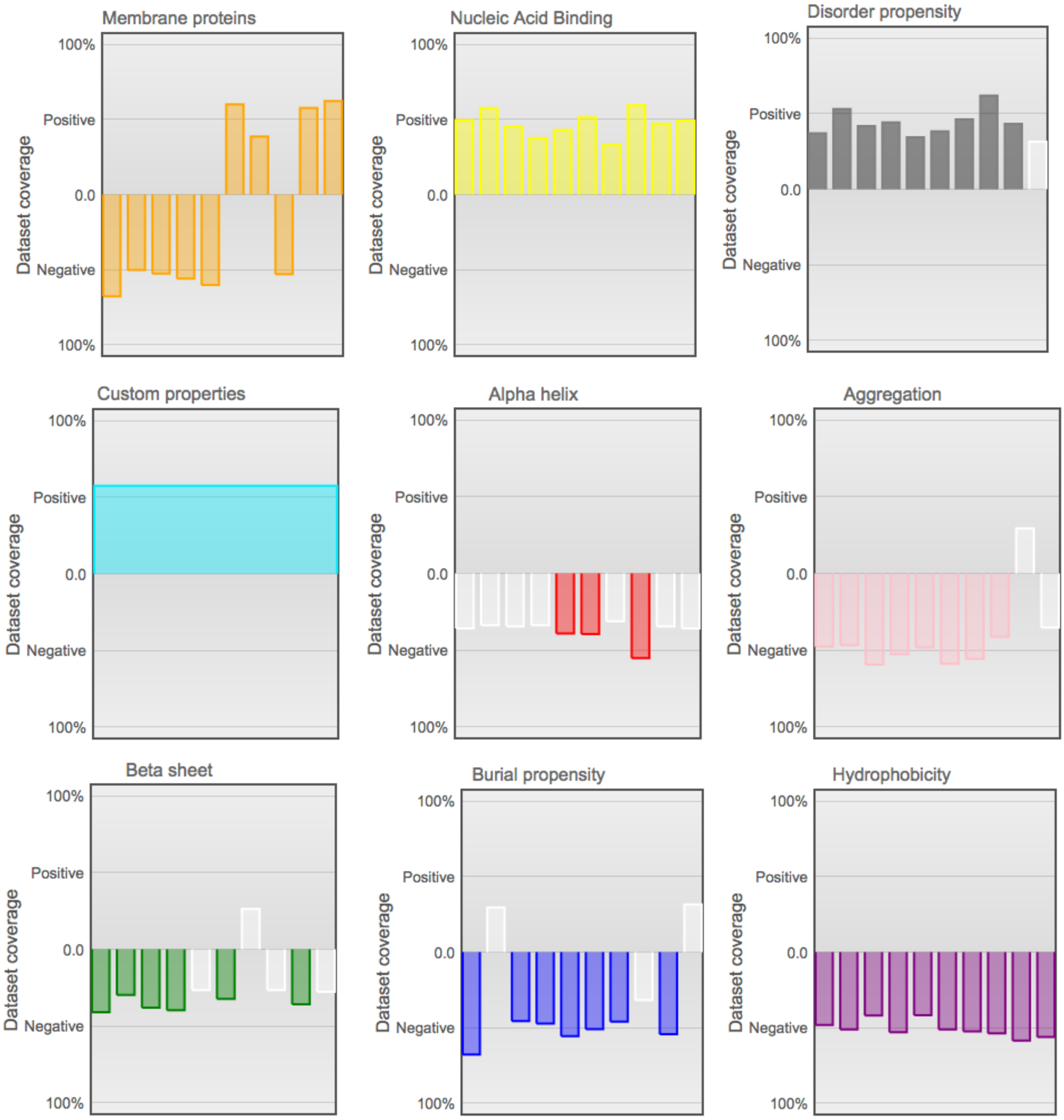

Supplementary Figure 5. Properties significantly different between ssRNA and dsRNA binders. Comparison made with cleverMachine[3]. The parameters for the plot using "custom properties" are taken from the polarity scale from *Chou* et al [2].

The data to build this image were extracted from our predictions[3] available at the webpage http://crg-webservice.s3.amazonaws.com/submissions/2018-04/124818/output/index.html?unlock=a798e0e111.

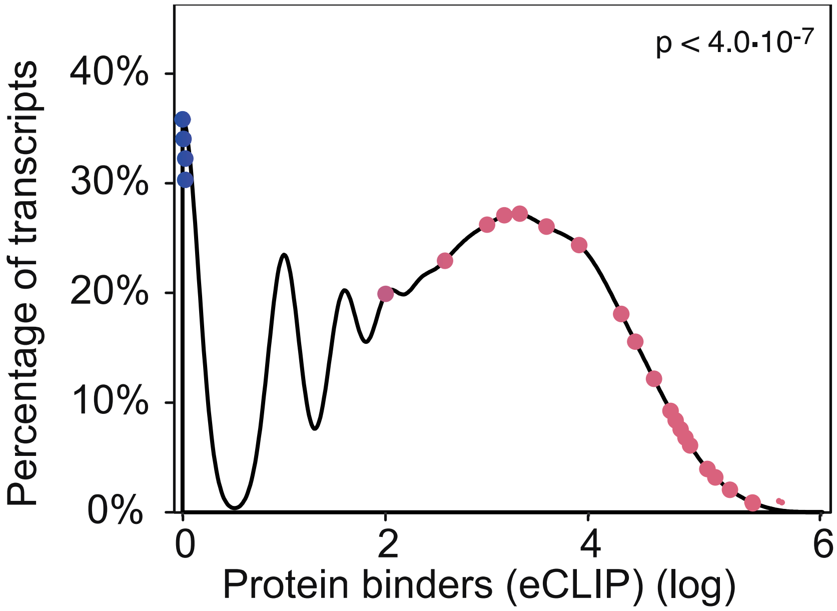

Supplementary Figure 6. RNAs coding for chaperones and RBP interactions. Fraction of proteins binding to RNA coding for chaperones as measured by eCLIP. The transcripts are represented as blue or pink dots according to their structural content (blue = low structural content and pink = high structural content; $p = 4x10^{-7}$; Kolmogorov-Smirnov).

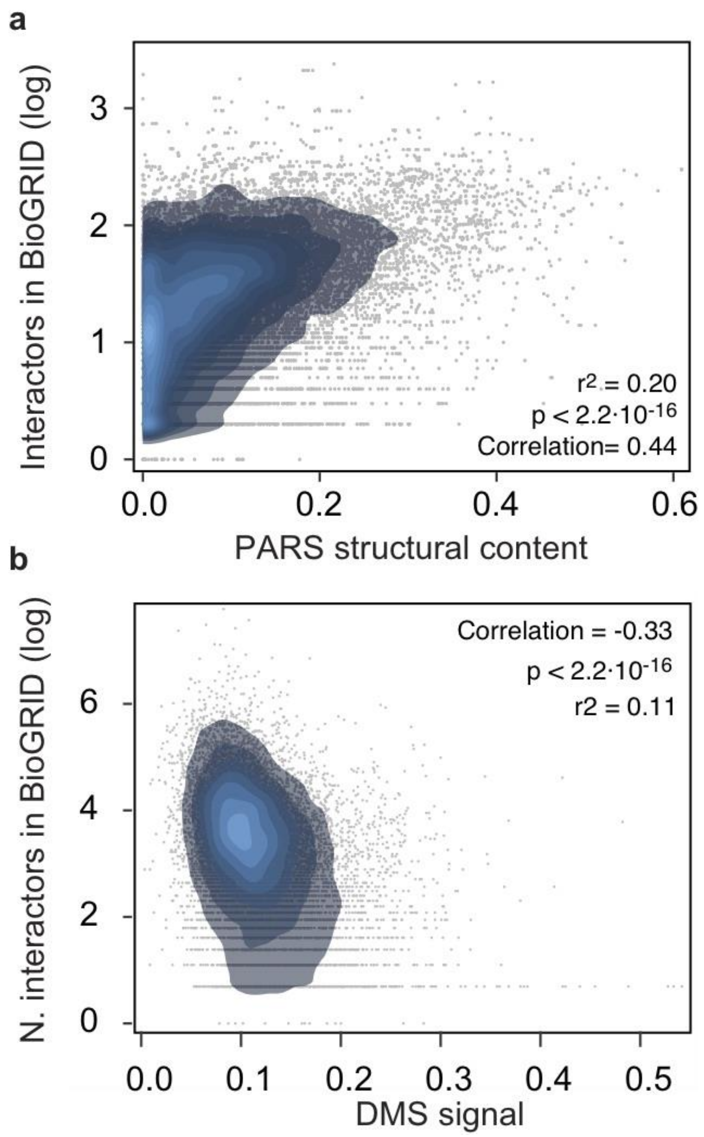

Supplementary Figure 7. Relationship between RNA secondary structure and the BioGRID reported interactors of the encoded proteins. a) Correlation between transcriptome PARS structural content and protein-protein interactions of the encoded product (BioGRID). **b**) Correlation between transcriptome DMS structural content and protein-protein interactions of the encoded product (BioGRID).

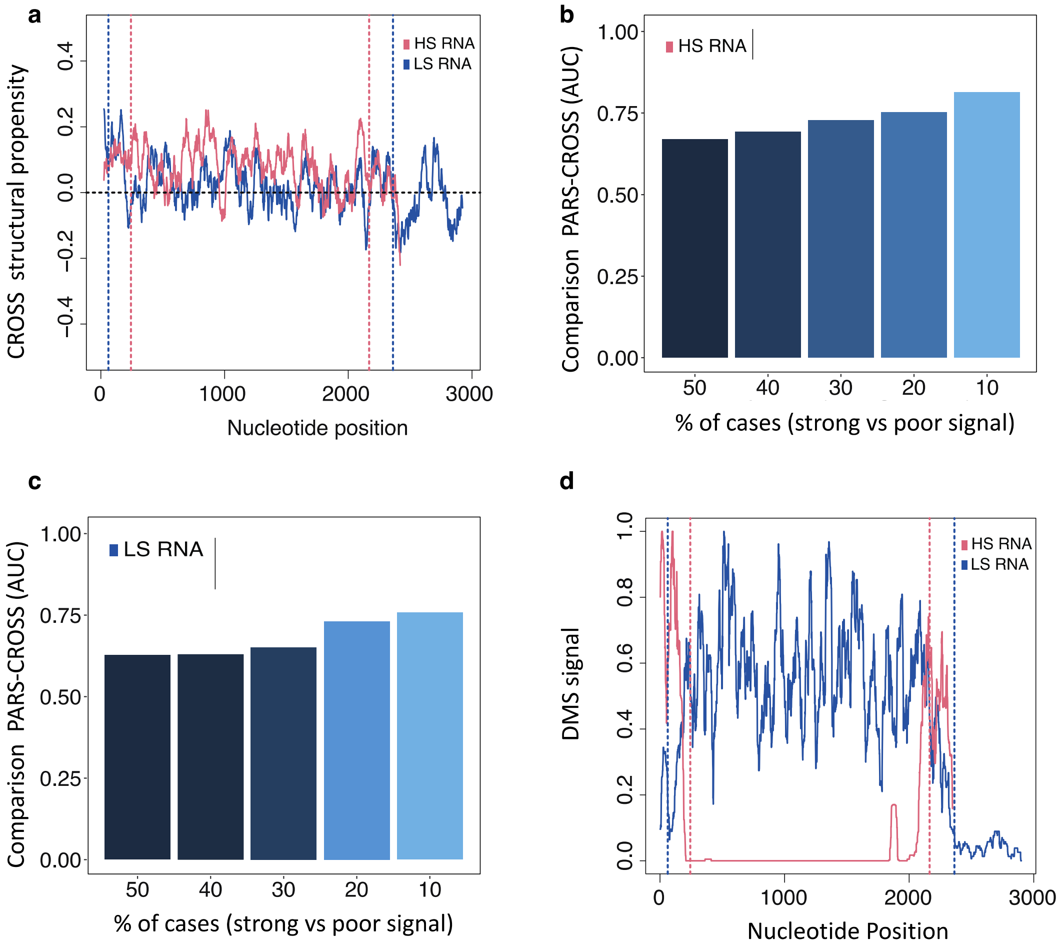

Supplementary Figure 8. CROSS and DMS structural content of HS and LS RNA. a) CROSS icSHAPE predicts that HS (*HSP70*) is more structured than LS (*BRaf*) RNA, in agreement with PARS experiments. Vertical dashed lines indicate UTRs; **b-c**) Comparison between PARS and CROSS icSHAPE for both **b**) HS and **c**) LS structures indicate strong agreement between predictions and experiments. From poor (50%) to strong (10%) PARS signals, the Area Under the ROC Curve (AUC) is used to assess CROSS performances on equal-size groups of nucleotides ranked by PARS scores. **d**) DMS signal analysis indicates that HS (*HSP70*) is more structured than LS (*BRaf*), in agreement with PARS experiments and CROSS predictions.

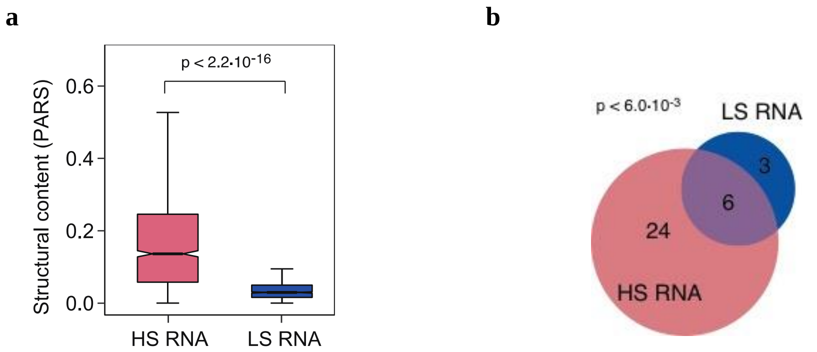

Supplementary Figure 9. *cat*RAPID and eCLIP data including UTRS. a) Boxplot distribution of the PARS the secondary structure content. **b**) Venn diagram showing the overlap (empirical p-value $p < 6{\cdot}10^{-3}$ computed on all the 100 eCLIP RBPs as background) between protein interactions of *HSP70* (HS, pink) and *BRaf* RNA (LS, blue).

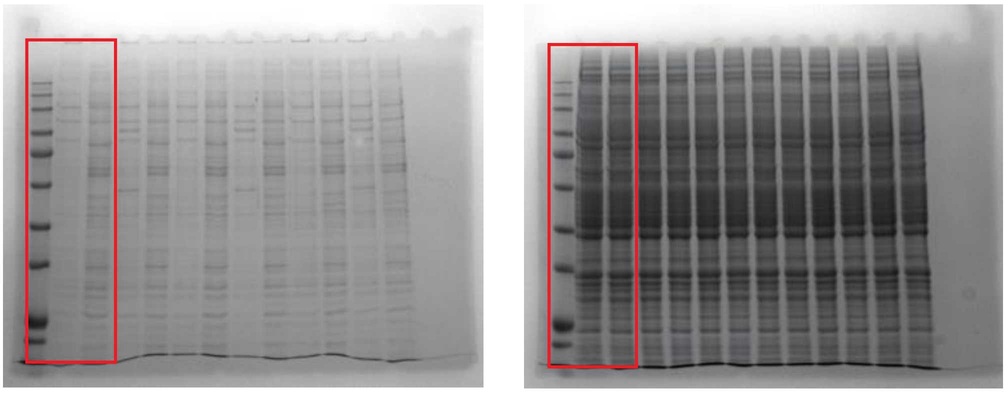

Supplementary Figure 10. Uncropped gels from Figure 4a. Coomassie blue-stained gels showing the protein separation upon the b-isox-driven aggregation. The red square marks the cropped section presented in Figure 4a.

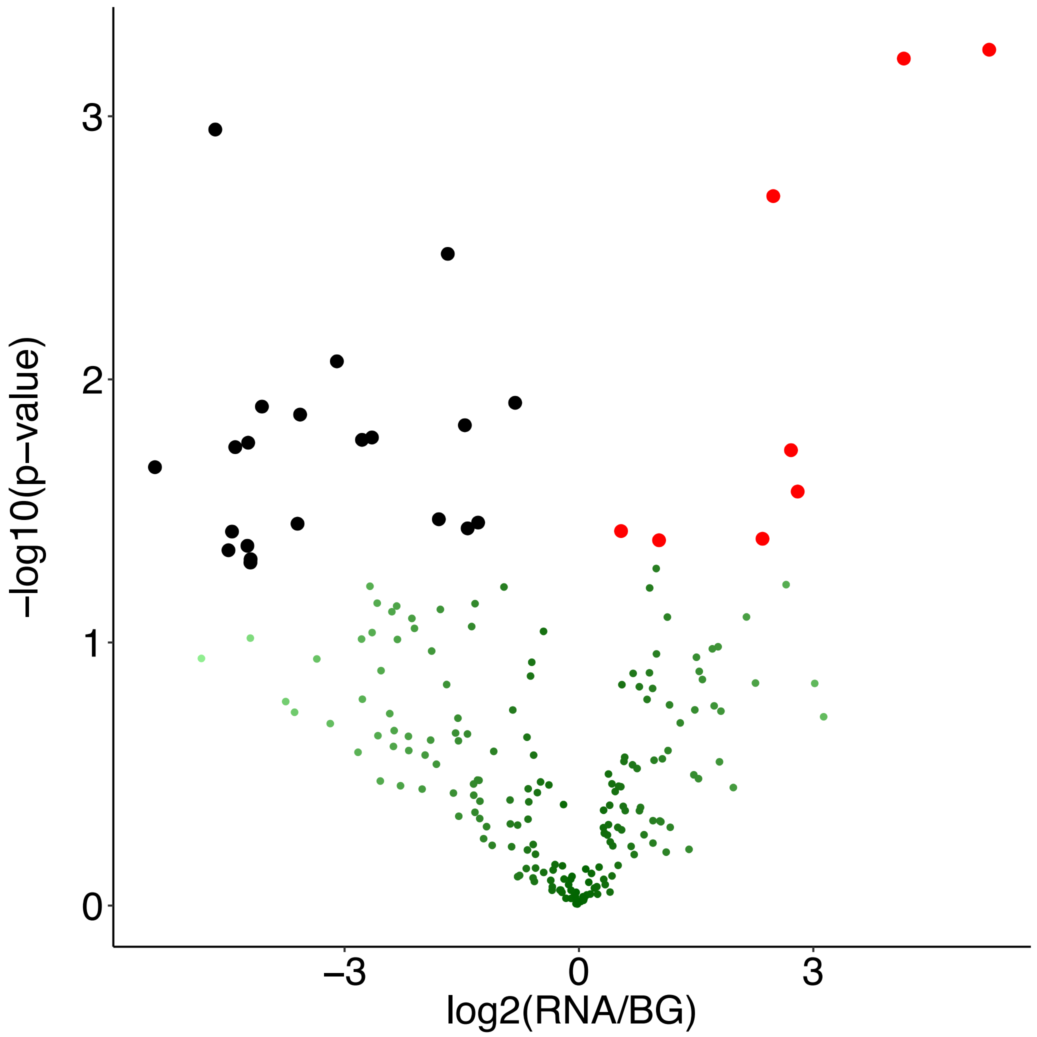

Supplementary Figure 11. Stringency of *HSP70* experiments. By reducing the RNA/Background ratio the number of false negatives decreases (see **Methods**). At low RNA/Background ratio we observe an increase in catRAPID predictive power (**Figure 5b**) (Area under the ROC curve) supporting our observation that released proteins (black dots) are direct binders of HSP70. In the plot, red dots represent proteins with increased abundance upon HSP70 incubation.

	Median DMS	Median PARS	Binds mainly to	Location	Ref.
**coding**	0.113	0.049	Proteins	Cytoplasm	[4, 5]
**snRNA**	0.112	0.032	Proteins	Nucleus	[6, 7, 8]
**snoRNA**	0.195	0.035	Proteins	Nucleolus	[9, 10]
**lincRNA**	0.150	0.001	Proteins and nucleotides	Cytoplasm/Nucleus	[11]
**scaRNA**	0.308	0.026	Proteins and nucleotides	Nucleus	[12]
**antisense**	0.172	0.002	Nucleotides	Cytoplasm	[13, 14, 15]
**miRNA**	0.360	0.010	Nucleotides	Cytoplasm	[16, 17]

Supplementary Table 1. Structural content by RNA type. secondary structure content measured by DMS and PARS arranged by the sum of the means max/min normalised.

**Property**	**Correlation**	**Ref.**
Hydrophobicity	-0.94	Fauchere *et al.*[18]
Hydrophobicity	-0.92	Roseman [19]
Hydrophobicity	-0.90	Black & Mould [20]
Hydrophobicity	-0.90	Sweet & Eisenberg [21]
Hydrophobicity	-0.86	Kyte & Doolittle [22]
Hydrophobicity	-0.83	Abraham & Leo [23]
Hydrophobicity	-0.82	Eisenberg *et al.*[24]
Hydrophobicity	-0.82	Bull & Breese [25]
Hydrophobicity	-0.82	Rao & Argos [26]
Hydrophobicity	-0.77	Janin [27]
Disorder	0.69	Campen *et al.* [28]

Supplementary Table 2. Correlation between polarity[29] and hydrophobicity or disorder.

Domain	Topology	Structure involved in the recognition	Chain recognised	Ref.
RRM	αβ	beta	ssRNA	[30]
KH	αβ	beta and loop	ssRNA	[31, 32]
dsRBD	αβ	alpha and loop	dsRNA	[33]
ZnF-CCHH	αβ	alpha	dsRNA	[34]
S1	β	beta	ssDNA	[35, 36]
PAZ	αβ	beta-barrel and alpha-beta	ssRNA	[37, 38, 39]
PIWI	αβ	alpha main pocket	dsRNA	[39, 40]
TRAP	β	beta	ssDNA	[41]
Pumilio	α	alpha	Structured RNA	[42, 43]
SAM	α	alpha	RNA hairpins	[44]

Supplementary Table 3. Common RNA-binding domains. Common RNA-binding domains, their structural elements and the type of RNA that they bind. The classification of proteins was taken from Lunde *at al.*[45] NA = Nucleic Acids. The table was adapted by permission from Springer Nature Customer Service Centre GmbH: Springer Nature, Nature Reviews Molecular Cell Biology, RNA-binding proteins: modular design for efficient function, Bradley M. Lunde, Claire Moore, Gabriele Varani, Copyright © 2007, Springer Nature (2007).

Supplementary References

1. Klus P, Ponti RD, Livi CM, Tartaglia GG. Protein aggregation, structural disorder and RNA-binding ability: a new approach for physico-chemical and gene ontology classification of multiple datasets. *BMC Genomics* **16**, 1071 (2015).

2. Chou PY, Fasman GD. Prediction of the secondary structure of proteins from their amino acid sequence. *Adv Enzymol Relat Areas Mol Biol* **47**, 45-148 (1978).

3. Klus P, Bolognesi B, Agostini F, Marchese D, Zanzoni A, Tartaglia GG. The cleverSuite approach for protein characterization: predictions of structural properties, solubility, chaperone requirements and RNA-binding abilities. *Bioinformatics* **30**, 1601-1608 (2014).

4. Faure G, Ogurtsov AY, Shabalina SA, Koonin EV. Role of mRNA structure in the control of protein folding. *Nucleic Acids Res* **44**, 10898-10911 (2016).

5. Sampath K, Ephrussi A. CncRNAs: RNAs with both coding and non-coding roles in development. *Development* **143**, 1234-1241 (2016).

6. Madhani HD. snRNA catalysts in the spliceosome's ancient core. *Cell* **155**, 1213-1215 (2013).

7. Montemayor EJ, Didychuk AL, Yake AD, Sidhu GK, Brow DA, Butcher SE. Architecture of the U6 snRNP reveals specific recognition of 3'-end processed U6 snRNA. *Nat Commun* **9**, 1749 (2018).

8. Liu S*, et al.* Binding of the human Prp31 Nop domain to a composite RNA-protein platform in U4 snRNP. *Science* **316**, 115-120 (2007).

9. Granneman S, Kudla G, Petfalski E, Tollervey D. Identification of protein binding sites on U3 snoRNA and pre-rRNA by UV cross-linking and high-throughput analysis of cDNAs. *Proc Natl Acad Sci U S A* **106**, 9613-9618 (2009).

10. Tomasevic N, Peculis B. Identification of a U8 snoRNA-specific binding protein. *J Biol Chem* **274**, 35914-35920 (1999).

11. Ulitsky I, Bartel DP. lincRNAs: genomics, evolution, and mechanisms. *Cell* **154**, 26-46 (2013).

12. Patil P*, et al.* scaRNAs regulate splicing and vertebrate heart development. *Biochim Biophys Acta* **1852**, 1619-1629 (2015).

13. Pelechano V, Steinmetz LM. Gene regulation by antisense transcription. *Nat Rev Genet* **14**, 880-893 (2013).

14. Saberi F, Kamali M, Najafi A, Yazdanparast A, Moghaddam MM. Natural antisense RNAs as mRNA regulatory elements in bacteria: a review on function and applications. *Cell Mol Biol Lett* **21**, 6 (2016).

15. Magistri M, Faghihi MA, St Laurent G, 3rd, Wahlestedt C. Regulation of chromatin structure by long noncoding RNAs: focus on natural antisense transcripts. *Trends Genet* **28**, 389-396 (2012).

16. Chapman EJ, Carrington JC. Specialization and evolution of endogenous small RNA pathways. *Nat Rev Genet* **8**, 884-896 (2007).

17. Carthew RW, Sontheimer EJ. Origins and Mechanisms of miRNAs and siRNAs. *Cell* **136**, 642-655 (2009).

18. Pliska J-LFaV. Hydrophobic parameters of pi amino-acid side chains from the partitioning of N-acetyl-amino-acid amides. *European Journal of Medicinal Chemistry* **4**, 369-375 (1983).

19. Roseman MA. Hydrophobicity of the peptide C=O...H-N hydrogen-bonded group. *J Mol Biol* **201**, 621-623 (1988).

20. Black SD, Mould DR. Development of hydrophobicity parameters to analyze proteins which bear post- or cotranslational modifications. *Anal Biochem* **193**, 72-82 (1991).

21. Sweet RM, Eisenberg D. Correlation of sequence hydrophobicities measures similarity in three-dimensional protein structure. *J Mol Biol* **171**, 479-488 (1983).

22. Kyte J, Doolittle RF. A simple method for displaying the hydropathic character of a protein. *J Mol Biol* **157**, 105-132 (1982).

23. Abraham DJ, Leo AJ. Extension of the fragment method to calculate amino acid zwitterion and side chain partition coefficients. *Proteins* **2**, 130-152 (1987).

24. Eisenberg D, Schwarz E, Komaromy M, Wall R. Analysis of membrane and surface protein sequences with the hydrophobic moment plot. *J Mol Biol* **179**, 125-142 (1984).

25. Bull HB, Breese K. Surface tension of amino acid solutions: a hydrophobicity scale of the amino acid residues. *Arch Biochem Biophys* **161**, 665-670 (1974).

26. Mohana Rao JK, Argos P. A conformational preference parameter to predict helices in integral membrane proteins. *Biochim Biophys Acta* **869**, 197-214 (1986).

27. Janin J. Surface and inside volumes in globular proteins. *Nature* **277**, 491-492 (1979).

28. Campen A, Williams RM, Brown CJ, Meng J, Uversky VN, Dunker AK. TOP-IDP-scale: a new amino acid scale measuring propensity for intrinsic disorder. *Protein Pept Lett* **15**, 956-963 (2008).

29. Grantham R. Amino acid difference formula to help explain protein evolution. *Science* **185**, 862-864 (1974).

30. Oubridge C, Ito N, Evans PR, Teo CH, Nagai K. Crystal structure at 1.92 A resolution of the RNA-binding domain of the U1A spliceosomal protein complexed with an RNA hairpin. *Nature* **372**, 432-438 (1994).

31. Beuth B, Pennell S, Arnvig KB, Martin SR, Taylor IA. Structure of a Mycobacterium tuberculosis NusA-RNA complex. *EMBO J* **24**, 3576-3587 (2005).

32. Lewis HA*, et al.* Sequence-specific RNA binding by a Nova KH domain: implications for paraneoplastic disease and the fragile X syndrome. *Cell* **100**, 323-332 (2000).

33. Ramos A*, et al.* RNA recognition by a Staufen double-stranded RNA-binding domain. *EMBO J* **19**, 997-1009 (2000).

34. Lu D, Searles MA, Klug A. Crystal structure of a zinc-finger-RNA complex reveals two modes of molecular recognition. *Nature* **426**, 96-100 (2003).

35. Liu Q, Greimann JC, Lima CD. Reconstitution, activities, and structure of the eukaryotic RNA exosome. *Cell* **127**, 1223-1237 (2006).

36. Frazao C*, et al.* Unravelling the dynamics of RNA degradation by ribonuclease II and its RNA-bound complex. *Nature* **443**, 110-114 (2006).

37. Macrae IJ*, et al.* Structural basis for double-stranded RNA processing by Dicer. *Science* **311**, 195-198 (2006).

38. Ma JB, Ye K, Patel DJ. Structural basis for overhang-specific small interfering RNA recognition by the PAZ domain. *Nature* **429**, 318-322 (2004).

39. Song JJ, Smith SK, Hannon GJ, Joshua-Tor L. Crystal structure of Argonaute and its implications for RISC slicer activity. *Science* **305**, 1434-1437 (2004).

40. Ma JB, Yuan YR, Meister G, Pei Y, Tuschl T, Patel DJ. Structural basis for 5'-end-specific recognition of guide RNA by the A. fulgidus Piwi protein. *Nature* **434**, 666-670 (2005).

41. Antson AA, Dodson EJ, Dodson G, Greaves RB, Chen X, Gollnick P. Structure of the trp RNA-binding attenuation protein, TRAP, bound to RNA. *Nature* **401**, 235-242 (1999).

42. Auweter SD, Oberstrass FC, Allain FH. Sequence-specific binding of single-stranded RNA: is there a code for recognition? *Nucleic Acids Res* **34**, 4943-4959 (2006).

43. Wang X, McLachlan J, Zamore PD, Hall TM. Modular recognition of RNA by a human pumilio-homology domain. *Cell* **110**, 501-512 (2002).

44. Aviv T, Lin Z, Ben-Ari G, Smibert CA, Sicheri F. Sequence-specific recognition of RNA hairpins by the SAM domain of Vts1p. *Nat Struct Mol Biol* **13**, 168-176 (2006).

45. Lunde BM, Moore C, Varani G. RNA-binding proteins: modular design for efficient function. *Nat Rev Mol Cell Biol* **8**, 479-490 (2007).
