# Supplementary material for: RNA structure drives interaction with proteins
Source: Nat Commun. 2019 Jul 19;10:3246. doi: 10.1038/s41467-019-10923-5 (PMC6642211; doi:10.1038/s41467-019-10923-5)
Supplement: Supplementary file 3 — Reporting Summary [file 41467_2019_10923_MOESM3_ESM.pdf]

nature research

Corresponding author(s): Gian Gaetano Tartaglia, R. Martin Vabulas

Last updated by author(s): May 24, 2019

Reporting Summary

Nature Research wishes to improve the reproducibility of the work that we publish. This form provides structure for consistency and transparency in reporting. For further information on Nature Research policies, see Authors & Referees and the Editorial Policy Checklist.

Statistics

For all statistical analyses, confirm that the following items are present in the figure legend, table legend, main text, or Methods section.

n/a	Confirmed	
☐	☒	The exact sample size (*n*) for each experimental group/condition, given as a discrete number and unit of measurement
☒	☐	A statement on whether measurements were taken from distinct samples or whether the same sample was measured repeatedly
☐	☒	The statistical test(s) used AND whether they are one- or two-sided<br>*Only common tests should be described solely by name; describe more complex techniques in the Methods section.*
☒	☐	A description of all covariates tested
☐	☒	A description of any assumptions or corrections, such as tests of normality and adjustment for multiple comparisons
☐	☒	A full description of the statistical parameters including central tendency (e.g. means) or other basic estimates (e.g. regression coefficient) AND variation (e.g. standard deviation) or associated estimates of uncertainty (e.g. confidence intervals)
☐	☒	For null hypothesis testing, the test statistic (e.g. *F*, *t*, *r*) with confidence intervals, effect sizes, degrees of freedom and *P* value noted<br>*Give P values as exact values whenever suitable.*
☒	☐	For Bayesian analysis, information on the choice of priors and Markov chain Monte Carlo settings
☒	☐	For hierarchical and complex designs, identification of the appropriate level for tests and full reporting of outcomes
☐	☒	Estimates of effect sizes (e.g. Cohen's *d*, Pearson's *r*), indicating how they were calculated

Our web collection on statistics for biologists contains articles on many of the points above.

Software and code

Policy information about availability of computer code

Data collection

catRAPID, CROSS, free for academic purposes, are available at www.tartaglialab.com.

RPISeq 11 is a sequence-based predictor of protein-RNA interactions based on two distinct classifiers, Support Vector Machine (SVM) and Random Forest (RF).

The versions used are referenced in the manuscript:The full list of protein-RNA interactions calculated with catRAPID is available in the RNAct database 14,33,34.

catRAPID omics was used to compute the interaction propensities of 100 LS and HS against the human RNA-binding sub-proteome 33. catRAPID omics uses large pre-compiled protein libraries and ranks interactions based on the propensity score, with strong predictive power as well as presence of motifs and RBDs 14.

We used catRAPID omiXcore 70 to calculate the interaction propensities of HSP70 mRNA with the ad hoc set of proteins present in the b-isox precipitate.We also used RPIseq.

We predicted the secondary structure of transcripts using CROSS (Computational Recognition of Secondary Structure) 39.

11   Muppirala, U. K., Honavar, V. G. & Dobbs, D. Predicting RNA-protein interactions using only sequence information. BMC Bioinformatics 12, 489, doi:10.1186/1471-2105-12-489 (2011).
14   Bellucci, M., Agostini, F., Masin, M. & Tartaglia, G. G. Predicting protein associations with long noncoding RNAs. Nat Methods 8, 444-445, doi:10.1038/nmeth.1611 (2011).
33   Agostini, F. et al. catRAPID omics: a web server for large-scale prediction of protein-RNA interactions. Bioinformatics 29, 2928-2930, doi:10.1093/bioinformatics/btt495 (2013).
34   Lang, B., Armaos, A. & Tartaglia, G. G. RNAct: Protein-RNA interaction predictions for model organisms with supporting experimental data. Nucleic acids research, doi:10.1093/nar/gky967 (2018).
39   Delli Ponti, R., Marti, S., Armaos, A. & Tartaglia, G. G. A high-throughput approach to profile RNA structure. Nucleic acids research

45, e35, doi:10.1093/nar/gkw1094 (2017).
70 Armaos, A., Cirillo, D. & Gaetano Tartaglia, G. omiXcore: a web server for prediction of protein interactions with large RNA. Bioinformatics 33, 3104-3106, doi:10.1093/bioinformatics/btx361 (2017).

Data analysis

To investigate functional differences between highly and poorly structured RNAs, we analysed GO terms associated to the least and most structured RNAs (100 LS vs 100 HS transcripts) using the cleverGO 36 approach.36 Klus, P., Ponti, R. D., Livi, C. M. & Tartaglia, G. G. Protein aggregation, structural disorder and RNA-binding ability: a new approach for physico-chemical and gene ontology classification of multiple datasets. BMC Genomics 16, 1071, doi:10.1186/s12864-015-2280-z (2015).

For manuscripts utilizing custom algorithms or software that are central to the research but not yet described in published literature, software must be made available to editors/reviewers. We strongly encourage code deposition in a community repository (e.g. GitHub). See the Nature Research guidelines for submitting code & software for further information.

Data

Policy information about availability of data

All manuscripts must include a data availability statement. This statement should provide the following information, where applicable:

- Accession codes, unique identifiers, or web links for publicly available datasets
- A list of figures that have associated raw data
- A description of any restrictions on data availability

The data that support the findings of this study are available from the corresponding author upon request. The mass spectrometry data (Figure 4) are available via ProteomeXchange with identifier PXD011751. In the figshare repository with DOI https://doi.org/10.6084/m9.figshare.c.4505759.v2 we report: (i) the physicochemical properties identified by multicleverMachine57, 74 to discriminate ssRNA and dsRNA binders from RNA binding proteins (RBPs); (ii) the physicochemical properties selected by the cleverMachine57 that discriminate ssRNA and dsRNA binders; (iii) the sets and results for the Gene Ontology and term analysis obtained with cleverGO37.

Field-specific reporting

Please select the one below that is the best fit for your research. If you are not sure, read the appropriate sections before making your selection.

☒ Life sciences ☐ Behavioural & social sciences ☐ Ecological, evolutionary & environmental sciences

For a reference copy of the document with all sections, see nature.com/documents/nr-reporting-summary-flat.pdf

Life sciences study design

All studies must disclose on these points even when the disclosure is negative.

Sample size	Lysates from 10 million cells were compared per condition. No additional methods were needed to pre-determine sample size.
Data exclusions	No data were excluded from the analysis.
Replication	All repetitions were independent biological repetitions performed four times.
Randomization	No randomization was used.
Blinding	No blinding was used during data collection and analyzes.

Reporting for specific materials, systems and methods

We require information from authors about some types of materials, experimental systems and methods used in many studies. Here, indicate whether each material, system or method listed is relevant to your study. If you are not sure if a list item applies to your research, read the appropriate section before selecting a response.

Materials & experimental systems

n/a	Involved in the study	
☒	☐	Antibodies
☐	☒	Eukaryotic cell lines
☒	☐	Palaeontology
☒	☐	Animals and other organisms
☒	☐	Human research participants
☒	☐	Clinical data

Methods

n/a	Involved in the study	
☒	☐	ChIP-seq
☒	☐	Flow cytometry
☒	☐	MRI-based neuroimaging

Eukaryotic cell lines

Policy information about cell lines

Cell line source(s)	HeLa cell line used in this study was from ATCC #CCL-2.
Authentication	HeLa cells used in this study were not authenticated.
Mycoplasma contamination	HeLa cells have been tested and found negative for mycoplasma contamination.
Commonly misidentified lines (See ICLAC register)	No commonly misidentified lines were used in this study.
