# Supplementary material for: RNA structure drives interaction with proteins
Source: Nat Commun. 2019 Jul 19;10:3246. doi: 10.1038/s41467-019-10923-5 (PMC6642211; doi:10.1038/s41467-019-10923-5)
Supplement: Supplementary file 4 — Description of Additional Supplementary Information [file 41467_2019_10923_MOESM4_ESM.pdf]

Description of Additional Supplementary Files

File Name: Supplementary Data 1
Description: List of protein and transcript pairs analysed with RPISeq and *cat*RAPID (linked to Supplementary Figure 1).

File Name: Supplementary Data 2
Description: List of RNA protein chain pairs (linked to Figure 1): PDB ID, RNA chain, protein chain, internal contacts (from Equation (2) of Methods) and external contacts (from Equation (3) of Methods) as well as PDB title

File Name: Supplementary Data 3
Description: List of Structural content (PARS) and number of protein binders (eCLIP) for both the HS and LS RNA sets.

File Name: Supplementary Data 4
Description: UniProt entries for exclusively single-stranded (ssRNA) and double-stranded RNA (dsRNA) binders.

File Name: Supplementary Data 5
Description: Protein enrichments in the b-isox aggregate: Protein ID, protein name, gene name, statistical significance (Student's test) and enrichment over the background for both *Hsp70* and *BRaf*.
